# Real Evidence and Misconceptions about Malignant Hyperthermia in Children: A Narrative Review

**DOI:** 10.3390/jcm12123869

**Published:** 2023-06-06

**Authors:** Luciano Frassanito, Fabio Sbaraglia, Alessandra Piersanti, Francesco Vassalli, Monica Lucente, Nicoletta Filetici, Bruno Antonio Zanfini, Stefano Catarci, Gaetano Draisci

**Affiliations:** 1 Department of Scienze dell’Emergenza, Anestesiologiche e della Rianimazione—IRCCS Fondazione Policlinico A. Gemelli, 00168 Rome, Italy; fabio.sbaraglia@policlinicogemelli.it (F.S.); alessandrapiersanti83@gmail.com (A.P.); monicalucente@gmail.com (M.L.); nicoletta.filetici@policlinicogemelli.it (N.F.); brunoantonio.zanfini@policlinicogemelli.it (B.A.Z.); stefano.catarci@policlinicogemelli.it (S.C.); gaetano.draisci@policlinicogemelli.it (G.D.); 2 Department of Critical Care and Perinatal Medicine, Istituto di Ricovero e Cura a Carattere Scientifico (IRCCS), Istituto Giannina Gaslini, 16147 Genoa, Italy; francescovasssalli@gmail.com

**Keywords:** malignant hyperthermia, pediatric anesthesia, general anesthesia, personalized medicine, genetic screening, patient safety, dantrolene

## Abstract

Malignant hyperthermia is a rare but life-threatening pharmacogenetic disorder triggered by exposure to specific anesthetic agents. Although this occurrence could affect virtually any patient during the perioperative time, the pediatric population is particularly vulnerable, and it has a five-fold higher incidence in children compared to adults. In the last few decades, synergistic efforts among leading anesthesiology, pediatrics, and neurology associations have produced new evidence concerning the diagnostic pathway, avoiding unnecessary testing and limiting false diagnoses. However, a personalized approach and an effective prevention policy focused on clearly recognizing the high-risk population, defining perioperative trigger-free hospitalization, and rapid activation of supportive therapy should be improved. Based on epidemiological data, many national scientific societies have produced consistent guidelines, but many misconceptions are common among physicians and healthcare workers. This review shall consider all these aspects and summarize the most recent updates.

## 1. Introduction

Since the beginning of the 20th century, cases of increased body temperature related to general anesthesia (GA) have been observed, and several reports referred to complications and anesthesia-related deaths, often described as “ether convulsions” [1,2].

Malignant hyperthermia (MH) is a rare but life-threatening heterogeneous pharmacogenetic disorder due to the dysfunction of skeletal muscle calcium channels, triggered by exposure to volatile halogenated anesthetics (desflurane, isoflurane, sevoflurane, halothane) and the depolarizing muscle relaxant succinylcholine, which results in abnormal contraction and a hypermetabolic state at the level of the myocyte that rapidly leads to ATP depletion, rhabdomyolysis, and heat production [3,4,5,6]. It was first described in 1962 as cases occurring in a single family by Denborough et al., when the authors reported a 21-year-old student who, when admitted to the Royal Melbourne Hospital in Australia for a leg fracture, was more concerned about receiving GA than about his fracture because 10 of his family members had died during or after GA, usually administered for minor procedures [3].

## 2. Epidemiology

Many physicians may believe that MH is so rare that most professionals will probably never face a case. However, there is a wide-ranging estimate of MH incidence [7]. The actual frequency of perioperative MH is challenging to estimate due to reluctance to publish adverse events, frequent misdiagnosis, and even poor adherence to pharmacovigilance registries [6,7,8,9]. Although most reported MH crises (>80%) occur in phenotypically normal children without a family history of MH-related comorbidities, we know that malignant hyperthermia susceptibility (MHS) is an autosomal dominant genetic condition that shows incomplete penetrance and affects all ethnic groups [6,7]. It is more common in males than females (2:1), with an estimated incidence of 1:10,000 in children and 1:50,000 in adults [10]. Indeed, MH mainly affects young patients, with a mean age of 18.3 years, probably due to increased penetrance in children, which, in turn, varies according to a specific genetic variant [6,10,11,12]. A higher concentration of MH-susceptible families is reported in Wisconsin and the upper Midwest in the United States [13].

## 3. Pathophysiology

**Figure 1:**
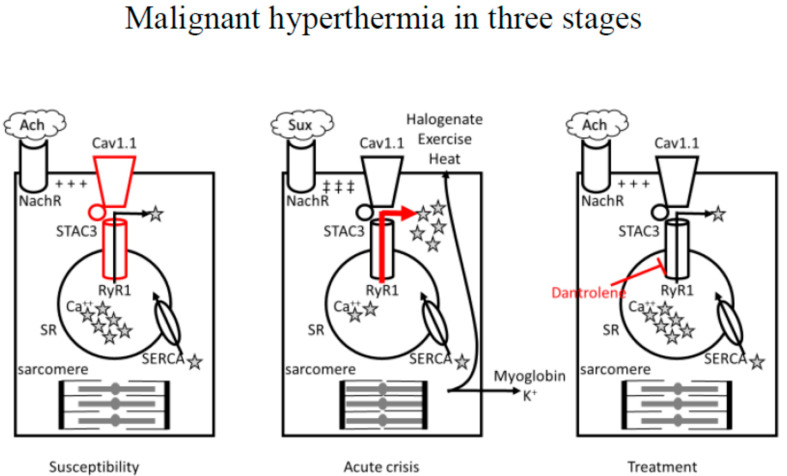
Role of dysfunction in the mechanism of excitation–contraction coupling in skeletal muscles primarily due to mutations in type 1 RyR1, Cav1.1, or STAC3 accessory protein in determining susceptibility to MH. Calcium release and uptake (arrows) is in equilibrium.

The root cause of MH is a dysfunction of excitation–contraction coupling in skeletal muscles, leading to excessive intracellular calcium (Ca^2+^) release (Figure 1).

In susceptible patients, exposure to halogenated volatile anesthetics (halogenate), depolarizing muscle relaxant succinylcholine, exercise, or heat could trigger an acute MH crisis characterized by excessive Ca^2+^ release (red arrow) from the sarcoplasmic reticulum with abnormal contraction and hypermetabolic state at the level of the myocyte that rapidly leads to ATP depletion, rhabdomyolysis, hyperkaliemia, and heat production. Dantrolene sodium, a post-synaptic muscle relaxant, can effectively revert an MH crisis by inhibiting RyR1-mediated intracellular calcium release from the sarcoplasmic reticulum of skeletal muscle cells (red blunted arrow).

RyR1: ryanodine receptor. Cav1.1: L-type voltage-gated calcium channel. MH: malignant hyperthermia. Ach: acetylcholine. NachR: nicotinic acetylcholine receptor. ★Ca^++^: calcium. Sux: succinylcholine. SERCA: sarcoendoplasmic reticulum calcium ATPase. ATP: adenosine triphosphate. SR: sarcoplasmic reticulum. +++: end-plate potential. ‡‡‡: prolonged end-plate potential.

Under physiologic conditions, following the release of acetylcholine at the neuromuscular junction, activation of the nicotinic receptor, and depolarization of the cell membrane, the dihydropyridine receptor (DHPR, also known as L-type voltage-gated calcium channel Cav1.1) on the T-tubular membrane is activated, and after direct interaction with Type 1 ryanodine receptor (RyR1) on the sarcoplasmic reticulum (SR) membrane, Ca^2+^ stored in the SR is released and becomes available for stimulation of the contractile apparatus [14]. Following muscle contraction, the sarcoendoplasmic reticulum calcium ATPase (SERCA) pump acts to transport Ca^2+^ from the cytosol back to the SR.

In MH, the leading abnormality is due to mutations in the gene RYR1, on chromosome 19q13.1, encoding for RyR1: missense mutations alter the receptor with gain-of-function mutations, inducing increased Ca^2+^ release into the cytoplasm [11]. Mutations in the gene RYR1 are also associated with three congenital myopathies and an isolated case of congenital myopathy characterized on histology by cores and rods [11]. More rarely, mutations in the α1 subunit of DHPR encoded by the gene CACNA1S may be involved: by suppressing the Ca^2+^ voltage-gated channel’s regulatory effect on RyR1, those variants can also cause an increased Ca^2+^ flux through the receptor [13,14]. Finally, mutations have been identified in the STAC3 accessory protein, required to correctly locate the calcium voltage-gated receptor within the skeletal muscle channel: those variants determine an increased amount of Ca^2+^ released in response to caffeine (a RyR1 agonist) and increase the amount of Ca^2+^ stored within the SR [11,12,13,14]. However, the paucity of clinical information surrounding the MH (or MH-like) episodes noted in patients with STAC3 variants, and the lack of robust experimental evidence in in vitro contracture testing, casts some doubt on an association between STAC3 variants and MHS [11,12,13,14,15,16,17,18].

### 3.1. Anesthetic-Induced MH

The most well-known risk factor for MH is the use of volatile anesthetic agents, such as halothane, isoflurane, sevoflurane, desflurane, and the depolarizing skeletal muscle relaxant succinylcholine [5,6,8]. Halothane-induced MH seems to contribute to most MH crises; however, of all volatile anesthetics, the prevalence of MH was highest when using sevoflurane [19]. Succinylcholine administered alone is reported to trigger adverse events in approximately 15.5% of MHS patients [20]. Combining inhaled anesthetic agents and succinylcholine can significantly increase the risk of MH. Although rare, one report suggested a triggering role for amide local anesthetics (lidocaine and bupivacaine), but it has never been confirmed [21]. Considering the broad use of local anesthetics in the susceptible population without new reports and the possibility of some unrecognized interaction with volatile agents, a relationship seems to be unlikely [22].

### 3.2. Non-Anesthetic Induced MH

MH may occur upon exposure to other factors, even in the absence of classic anesthetic triggering agents. Increasing evidence indicates that environmental heat stroke and exertion rhabdomyolysis caused by vigorous exercise and environmental heat can induce a life-threatening hyperthermic crisis in susceptible individuals [23,24,25]. The availability of an animal model of MH, as certain breeds of pigs were found by chance to be susceptible to this anesthetic complication and to have an underlying muscle disease, provided additional evidence [18]. It has been demonstrated that overheating alone can trigger fatal MH in susceptible experimental piglets, thus supporting the association between MHS and heat stroke in humans and between MHS and sudden infant death syndrome, which may be due to overheating [18]. Some patients with environmental heat stroke have been found to have histories or family histories of MH or an association among MH-related genetic defects [26,27,28,29]. A rare case of a non-anesthetic, stress-induced hyperpyrexia death was described in a 12-year-old male who experienced an MH crisis during a humerus fracture operation and 8 months later presented MH followed by sudden death after exertion [27]. Furthermore, emotional stress may also cause or contribute to stress-induced MH [30,31,32,33].

## 4. Disorders Associated with Malignant Hyperthermia

The literature recognizes several myopathies associated with MHS and suggests several others (Table 1) [34,35].

Motor neuron diseases are one of the most implicated in this context. For example, amyotrophic lateral sclerosis and spinal muscular atrophy involve the degeneration of motor neurons, thus causing weakness, muscle atrophy, and spasticity, and therefore do not confer an increased probability of MHS [35].

Myelin sheath disorders are another series of disorders that may cause weakness but do not directly affect the muscle fiber and therefore do not increase susceptibility to MH [36]. However, areas of demyelination may be more prone to toxicity by local anesthetics; thus, GA may be preferred [36].

Autoimmune disorders (myasthenia gravis and Lambert–Eaton syndrome) are characterized by the presence of pathogenic antibodies directed against the acetylcholine receptor at the neuromuscular junction, and although they may cause abnormal responses to depolarizing and non-depolarizing neuromuscular blocking agents, they do not increase MHS [36,37].

Similarly, dystrophic and non-dystrophic myotonic syndromes, a broad class of rare multisystemic myopathies clinically characterized by a combination of myotonia (impairment of muscle relaxation after voluntary contraction), muscle weakness, wasting, and myalgia due to genetic defects which involve the muscular isoforms of various ion channels, have traditionally, albeit erroneously, been considered at increased risk of developing MH [35]. Patients with these myopathies have a chance of developing MH that is equivalent to that of the general population, with one exception, represented by hypokalemic periodic paralysis (HypoPP), in most cases caused by mutations in the skeletal muscle voltage-gated Ca^2+^ channel encoded by CACNA1S (HypoPP type 1) [36]. However, the latest research shows that myotonic patients with MH crisis can have mutations at two distinct genetic loci, one for myotonia and one for MHS [14,38]. Therefore, although episodes occurred without evidence of the MH hallmark of hypermetabolism, non-triggering anesthetics should be recommended to reduce the risk of rhabdomyolysis [38].

The dystrophinopathies, which include Duchenne and Becker muscular dystrophy, cover a spectrum of X-linked muscle diseases, usually presenting in early childhood, characterized by progressive proximal muscle weakness and muscle fiber degeneration [37,39]. In addition, there are some concerns about the risk of MH because, in these patients, phenomena of volatile anesthetic-induced rhabdomyolysis and hyperkalemia are described [37,39].

A very challenging category of neuromuscular syndromes is that of mitochondrial diseases [40]. Kearns–Sayre syndrome, mitochondrial encephalomyopathy, lactic acidosis, stroke-like episodes (MELAS), or Leigh syndrome could be manifested in many overlapping patterns, but pediatric onset is generally more severe [40]. Because patients with mitochondrial diseases demonstrate hypersensitivity to volatile anesthetics, many practitioners avoid using volatile agents [41]. However, evidence denies any connection between mitochondrial disease and MH [42]. On the contrary halogenated agents characterized by rapid elimination (sevoflurane, desflurane) can be used, but caution should be paid to the increased risk of developing propofol-related infusion syndrome [42].

Some reports put rare or very rare diseases and metabolic syndromes under the MH spotlight, although the literature does not support suspicions in most cases. For example, glycogen storage diseases are not considered at risk of MH due to unclear metabolic adverse events described in some reports [43]. All anesthetic agents have been used for GA in children with Pompe disease, but no complication could be clearly associated with clinical MH [44]. Similarly, McArdle’s disease has been considered in the MH group for two patients testing positive for the in vitro contracture test and atypical reactions [44]. McArdle’s patients are probably more susceptible to muscle cramps, which may cause diagnostic confusion [44]. Patients with Noonan syndrome share a similar phenotypic appearance with King–Denborough syndrome, including pterygium colli, down-slanting palpebral fissures, eyelid ptosis, short stature, and pectus excavatum, and have long been associated with an increased risk of MH [45,46]. However, the absence of proof emerging from the literature, along with knowledge of the genetic basis of the 2 disorders (mutation on chromosome 19 near the gene that encodes the ryanodine receptor in the King–Denbourough syndrome and on chromosome 12 for the Noonan syndrome) led to the exclusion of MHS in Noonan syndrome patients [45,46].

Myopathies clearly recognized to be associated with MH are masseter muscle rigidity (MMR), central core disease (CCD), multi-mini core disease (MmD), centronuclear myopathy, HypoPP, Native American myopathy (NAM), and King–Denbourough syndrome [6,7,8,9,10,12,47].

Succinylcholine-induced MMR occurs in 1 in 100 children after induction with inhaled anesthesia and succinylcholine administration, and the clinical incidence of MH after MMR is estimated to be 15% [48,49,50]. However, muscle biopsy reveals that 50% of patients experiencing MMR show MHS [49]. CCD refers to a rare non-progressive myopathy caused by an RYR1 mutation with mainly autosomal dominant inheritance, presenting in infancy, characterized by hypotonia and muscle weakness, sustained by a predominance of type I muscular fibers containing clearly defined areas (cores) lacking oxidative enzyme activity [51,52,53,54]. The mutation of RYR1 in CCD implies insufficient Ca^2+^ concentration in the cytoplasm, causes excitation–contraction decoupling, and finally leads to clinical muscle weakness [53,54,55]. These patients often demonstrate MHS, but MH and CCD phenotypes do not always co-segregate within families [56,57]. Patients with MH may present with cores despite being clinically asymptomatic and with some RYR1 variants specific to CCD. Although RYR1 variants are the most commonly identified cause of CCD, they show genetic heterogeneity [56,57]. MmD is an autosomal recessive, early onset congenital myopathy that strikes bulbar, respiratory, and extraocular muscles [58]. Some variants of RYR1 resulting in altered Ca^2+^ release from intracellular stores have been associated with MmD [59]. Currently, the most accredited hypothesis is that one subset of RYR1 variants may result in both MH and MmD while another may be associated only with MmD [6,11].

## 5. Clinical Presentation

MH may occur at any time during anesthesia or in the early postoperative period, with variable progression and outcome. Clinical presentation in children is often insidious: hemodynamic responses, the precariousness of homeostatic equilibrium, and inadequate monitoring equipment could make a quick diagnosis more challenging. A retrospective analysis of the North American MH Registry and the Hiroshima University MH database revealed varying presentation, clinical course, and outcome based on the age group considered (0 to 24 months, 25 months to 12 years, and 13 to 18 years), mirroring the developmental changes in body structure and muscle composition throughout childhood [60,61]. Sinus tachycardia, hypercarbia despite increased minute ventilation, and rapid temperature increase are the most common findings in the pediatric population, together with masseter muscle spasm, especially in the middle age group; however, it is unclear whether the latter sign is due to physical characteristics or to the relatively high concentrations of volatile anesthetics frequently used for anesthesia induction in this age group [60]. A dramatic body temperature elevation is a common sign of MH reactions [62,63,64]. End-tidal carbon dioxide (ETCO_2_) is considered a sensitive early sign of MH, and rather than an abrupt rise in CO_2_, a more gradual rise is described [6,65]. Uncontrolled hypermetabolism is then followed by respiratory and metabolic acidosis due to rapid consumption of ATP, while rhabdomyolysis can result in life-threatening hyperkalemia, myoglobinuria, elevated creatine kinase, acute renal failure, arrhythmias, bowel ischemia, and compartment syndrome; disseminated intravascular coagulation could arise should the temperature exceed about 41 °C [15,62].

AMRA (adverse metabolic or muscular reaction to anesthesia) reports submitted to The North American Malignant Hyperthermia Registry of the Malignant Hyperthermia Association of the United States from 1987 to 2006 revealed a 1.4% death rate for 291 MH events and a 9.5% death rate for 84 US or Canadian reports of adverse events occurring between 2007 and 2012 in “very likely MH” or “almost certain MH” events [60,62,63]. In addition, the likelihood of any complication is reported to increase 2.9 times per 2 °C rise in maximum temperature [62].

Especially in children with a small body surface area, several conditions can enter into differential diagnosis with MH during anesthesia, including insufficient anesthesia or analgesia, insufficient ventilation, equipment malfunction, elevated end-tidal CO_2_ due to laparoscopic surgery, and iatrogenic overheating. In their absence, even events such as anaphylactic reactions, sepsis, thyroid storm, pheochromocytoma, transfusion reactions, ecstasy or other recreational drugs, neuroleptic malignant syndrome, and serotonin syndrome should be considered in the presence of MH suspicion. Knowledge of their clinical features and arterial and venous blood gas analysis is essential for correct diagnosis [3,4,7].

Drug-induced rhabdomyolysis should be considered in patients with neuromuscular diseases, while myotonic crises may occur in patients with myotonic syndromes after receiving succinylcholine or acetylcholinesterase inhibitors.

Another unusual situation at risk of MH is sedation with inhaled anesthetics in the pediatric intensive care unit (PICU) [66,67,68]. If these types of sedation devices are used, children susceptible to MH in the PICU may be at risk for such exposure, highlighting the significance of MH differential diagnosis in intensive care patients admitted for other conditions [69].

## 6. Clinical Diagnostic Pathway

**Figure 2:**
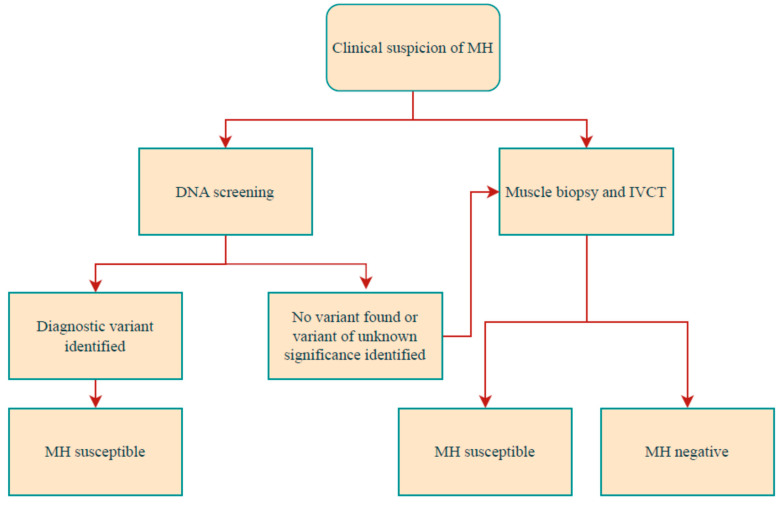
Diagnostic pathway for investigation of MH susceptibility.

Identifying the population at risk is of utmost importance to avoid an MH crisis. On the other hand, hastily applying an improper diagnosis of susceptibility could affect the medical future of an otherwise healthy child. Consequently, during preoperative evaluation, all children must be correctly screened (Figure 2).

The IVCT is recommended for individuals suspected to be at increased risk of MH either as a first-line test or when DNA analyses have failed to confirm the high-risk status. DNA screening on a patient’s blood sample is minimally invasive and affordable, but sensitivity is unfortunately only approximately 50% for the detection of MH susceptibility. If one of the known MH-associated mutations is identified, the subject should be considered at increased risk of developing MH. If DNA testing is negative, MHS still cannot be ruled out definitively, and the decision on the following diagnostic steps must then be based on the clinical indication. The decision to pursue either DNA screening or muscle biopsy and IVCT in the first instance will be made on a patient-by-patient basis by the MH diagnostic centre in consultation with the patient and their health-care funder, taking into consideration the availability of the respective tests, the urgency of the test, the prior probability of a positive diagnosis, and the costs of the tests in the relevant laboratory. The genetic laboratory is responsible for consulting the available published evidence (literature and databases) and applying prediction algorithms to eventually classify the variant as neutral or potentially MH-associated.

IVCT, in vitro contracture test; MH, malignant hyperthermia; MHS: malignant hyperthermia susceptibility.

Family history of undefined adverse events during surgery, unexplained perioperative death, postoperative or recurrent rhabdomyolysis, an idiopathic increase in creatine kinase levels, heat stroke, or rare diseases should be investigated [6,8,9]. In addition, previous exposure to halogenated anesthetics without reactions is no guarantee: even though an MH crisis may develop upon first GA with those agents, patients require an average of three exposures to anesthesia before triggering [6,8,9]. However, pre-operative creatine kinase screening is not recommended in the pediatric population [34,35,36].

The main red flag when evaluating children is muscle weakness because many neuromuscular diseases are associated with a high-risk population [34]. Questions should be addressed to investigate a family or personal history of congenital hip dislocation, motor stage delay, walking disorders, limb or limb strength defects, muscle fatigue, muscle pain, cramps sine causa, or frequent falls. Decreased muscle tone and/or trophism, expressionless myopathic facies, and ophthalmoplegia should raise suspicion of an underlying myopathy and be further investigated [34].

Due to multisystemic involvement, possible concomitant cardiomyopathies, arrhythmias, restrictive lung diseases, and endocrine and hepatorenal defects should also be carefully investigated in children suspected of congenital myopathy [15,16].

When evaluating a child with suspected MHS, parents should be informed and referred for investigation if anesthetic, medical, and family history cannot rule out an increased risk [47].

In the suspected but not overt group, allocation to the high-risk or standard-risk group could be challenging. Despite national recommendations, a clear diagnostic pathway must still be fully defined. Clinical signs should be accompanied by laboratory features confirming the suspicion.

The highest sensitivity test for detecting susceptibility to MH is a pharmacological challenge test performed on freshly excised skeletal muscle specimens under strictly controlled laboratory conditions, collectively referred to as the in vitro contracture test (IVCT) [47,70,71,72,73,74]. The IVCT is recommended for individuals suspected to be at increased risk of MH either as a first-line test or when DNA analyses have failed to confirm the high-risk status [47,70,71,72,73,74]. IVCT measures the contracture response to gradually increasing concentrations of caffeine and halothane and is referred to as the caffeine/halothane contracture test (CHCT) in North America and as the IVCT in Europe and elsewhere [70,71,72,73,74]. It is performed on a muscle biopsy of approximately 100–200 mg, measuring 20–25 mm in length with a thickness of 2–3 mm obtained from the vastus lateralis or medialis of patients ≥ 10 years old or ≥20 kg (in this case, parents can be tested instead) and differences among the two protocols essentially concern drug concentration and the number of muscles bundles that need to be tested for each drug [73]. Achieving the threshold concentration (producing a contracture of 2 mN or 0.2 g force) of halothane and caffeine, along with the tension produced at 2% halothane and 2 mM caffeine, confirms MHS diagnosis in Europe [34]. However, despite scientific society indications, there are very few centers able to provide the IVCT [12]; it is a highly invasive test (requiring minor surgery itself), expensive, and many doubts have been expressed in this regard [75].

DNA screening on a patient’s blood sample is minimally invasive and affordable, but sensitivity is unfortunately only approximately 50% for the detection of MHS; RYR1 targeted analysis of the known MH-associated mutations or screening of the entire coding regions is possible [70]. If one of the known MH-associated mutations is identified, the subject should be considered at increased risk of developing MH. If testing for an RYR1 mutation is negative, MHS still cannot be ruled out definitively, and the decision on the following diagnostic steps must then be based on the clinical indication. When the entire coding region of RYR1 is screened, yet-to-be-classified sequence variants may frequently be identified [70,72]. The genetic laboratory is responsible for consulting the available published evidence (literature and databases) and applying prediction algorithms to eventually classify the variant as neutral or potentially MH-associated. For patient safety, individuals carrying a likely pathogenic variant RYR1 variant should be regarded to be at increased risk for MH until further diagnostic tests, i.e., an IVCT, can be performed [47,72,76].

The decision to pursue either DNA screening or muscle biopsy and IVCT in the first instance should be made on a patient-by-patient basis by the MH diagnostic center (involving pediatric anesthesiologist) in consultation with the patient and their health-care funder, taking into consideration the availability of the respective tests, the urgency of the test, the prior probability of a positive diagnosis, and the costs of the tests in the relevant laboratory.

The EMHG, the MH Association of the United States (MHAUS), and the MH Group of Australia and New Zealand (MHANZ) are the worldwide largest multidisciplinary organizations promoting research and counseling on MH.

## 7. Perioperative Recommendations for High-Risk Children

Although it could be advisable to clarify the MH status of suspected patients before surgery, suspicion of MHS should not delay treatment of the surgical pathology, even in ambulatory settings, particularly if this would risk the progression of the condition of the patient, because the mere avoidance of triggering substances eliminates the risk of developing an MH event.

Trigger-free anesthesia should be performed, and careful monitoring of all vital signs, including EtCO_2_ and core temperature, should be adopted during the entire perioperative period [16]. It should also be emphasized that many of these anesthetics (such as halothane) are obsolete in modern anesthesia.

Regional anesthesia techniques (e.g., spinal, epidural, and peripheral nerve blocks) or total intravenous anesthesia (TIVA) are safe for these patients, while classical inhalational induction with halogenated agents must be avoided. This implies that peripheral venous access should be established before the onset of general anesthesia; in non-cooperative children, premedication with benzodiazepines, sedation with nitrous oxide, and topical local anesthetics can reduce the stress associated with needle punctures. Moreover, pediatric regional anesthesia is usually performed in combination with general anesthesia or some degree of sedation. There is no evidence to support elective intensive care unit management of MH-susceptible patients after uneventful trigger-free anesthesia [67].

In patients with congenital myopathies, premedication should be avoided or used with reduced dosage due to the increased sensitivity to sedatives and opioids and the risk of central respiratory depression, airway obstruction, and worsening of muscle weakness.

The non-depolarizing neuromuscular blocking agents may need a lower initial dose and could have a prolonged effect in patients with neuromuscular disorders; reversal with cholinesterase inhibitors before extubating is contraindicated.

Even the children affected by neuromuscular disorders, regardless of their association with MH, usually require a personalized anesthetic approach: succinylcholine and halogenated agents, the same drugs that can trigger MH in an MHS patient, can cause rhabdomyolysis in Duchenne syndrome and should therefore be avoided, while propofol infusions are best avoided in patients with mitochondrial diseases (boluses are commonly tolerated) [34,35]. Therefore, anesthesia for a child with undiagnosed myopathy may be a challenge. In addition, myopathy might not be clinically evident in the neonate requiring general anesthesia [34,35,36].

If available, a dedicated “vapor-free” machine for MH-susceptible patients is advisable, but if not, recommendations on anesthesia workstations must be followed. Since most vaporizers have a significant reservoir of the volatile anesthetic agent, they must be removed from the workstation when preparing it for use, and the breathing circuits should be replaced, as well as the soda lime canister. The circuit should be flushed with oxygen or air with maximal flow rate for workstation-specific time or, in the absence of manufacturer instructions, with a ventilatory pattern of 600 mL tidal volume and a ventilatory frequency of 15 min^−1^. This process will take some time and influence the operating room planning, often leading to scheduling the patient as the first intervention of the day. The anesthetic workstation should be flushed according to manufacturer guidelines; however, these recommendations are variable and may require two hours or more flushing. After the anesthesia machine has been flushed for the recommended time, it should not be set to standby mode before use.

Activated charcoal filters (ACFs) have been shown to rapidly and cost-efficiently decrease the concentration of anesthetic vapors to <5 ppm in 2–3 min and to maintain this low concentration during the course of general anesthesia for up to 12 h with fresh gas flows of at least 3 L min^−1^ and should be applied on both the inspiratory and expiratory branches of the breathing circuit [8]. If available, especially in the case of limited time before surgery, ACF could obviate the need for purging the system as described. However, the anesthesia machine will still need to be flushed with high fresh gas flows (≥10 L/min) for 90 s before placing the activated charcoal filters on both the inspiratory and expiratory branches of the breathing circuit [8,12].

Dedicated malignant hyperthermia charts or kits should also be implemented to reduce the response time in case of an acute crisis. Cognitive aids should be available in all operating rooms, and simulation of these rare clinical cases should be provided to active practitioners and residents.

## 8. Treatment of Malignant Hyperthermia

Since the first description of MH, it has been evident that discontinuing the anesthetic trigger and supportive measures could lead to the spontaneous resolution of the acute crisis [77]. Immediate management of suspected episodes includes avoiding succinylcholine administration, removing the vaporizer from the anesthetic circuit, washing the circuit with a maximal flow of 100% oxygen, and immediately transitioning to general anesthesia maintenance with non-triggering agents, typically TIVA [67]. Changing the breathing circuit, carbon dioxide absorber, or anesthesia workstation to accelerate the elimination of residual volatile halogenated agents is rational but time-consuming and discouraged in the first instance; an ACF inserted into the circuit could achieve the same objective more rapidly [47].

The team should be rapidly notified so that help can be called, dantrolene requested, and the surgery postponed or terminated as soon as possible. If not already performed, the airways should be secured with an endotracheal tube. Standard anesthetic monitoring, including pulse oximetry, ECG, non-invasive blood pressure, and EtCO_2_, should be completed with core temperature measurement; if not already in place, nasopharyngeal or low esophageal probes are typically a good compromise between accuracy and invasiveness, but alternative methods, such as tympanic, axillary, bladder, or skin temperature, can be considered [78]. The attending anesthesiologist should review the type and caliber of venous accesses, and large-bore venous lines should be secured early in the management; a central venous line should also be considered [34]. Arterial line and urinary catheter placement are usually performed, and blood samples for point-of-care testing and laboratory analysis should be drawn, but their relevance increases with time after immediate management. Dantrolene sodium, introduced in 1979, is a post-synaptic muscle relaxant that inhibits Ryr1-mediated intracellular calcium release from the sarcoplasmic reticulum of skeletal muscle cells and should be administered as soon as MH is suspected since it can effectively revert its pathophysiology [4]. Indeed, mortality of MH has precipitated from 80% to <5% [4,39]. The drug is approved by both the Food and Drug Administration and the European Medicines Agency for pediatric use [79,80]. The initial dantrolene dose for MH is 2–2.5 mg·kg^−1^ of actual body weight [81]. Ideal body weight calculations are avoided due to the risk of under-treatment; further boluses of 1–2.5 mg·kg^−1^ can be administered at a minimum of 10 min intervals and should be continued until a clinical response is observed, such as decreasing EtCO_2_ or PaCO_2_ for the same minute ventilation or normal EtCO_2_ or PaCO_2_ with normal minute ventilation, decreasing muscle rigidity, decreasing body temperature and lowering heart rate [81]. The maximum dose of 10 mg·kg^−1^ quoted in the official drug information can be exceeded if clinical stability is not achieved; doses up to 30 mg·kg^−1^ have been reported, although lack of clinical improvement after such high doses should prompt the clinician to consider alternate diagnoses [41].

A significant drawback of dantrolene sodium is related to its classical pharmaceutical formulation: Dantrium/Revonto/dantrolene sodium 20 mg of lyophilized orange powder needs to be diluted in 60 mL of sterile water (the drug will not completely dissolve in crystalloid-containing solutions) with a filter, leading to a final concentration of 0.32 mg/mL and a preparation time of about 3 min per vial. The water for diluting dantrolene should not be stored in a refrigerator; it may be stored in a warming cabinet designed to maintain fluid temperatures between 35 °C and 40 °C [82]. The number of vials varies widely according to the patient’s weight: while for a 10 kg infant, a single vial can provide the correct amount for the first bolus in 3 min, for a 50 kg pediatric patient, five vials and potentially 15 min are needed for the initial bolus to be administered. Since the time to intervention is critical in the prognosis of this emergency, newer formulations addressed this issue, but availability is suboptimal: RYANODEX^®^, 250 mg per vial to be reconstituted with 5 mL of sterile water (final concentration 50 mg/mL), is approved for the United States only, NPJ5008, nanoparticle formulation 120 mg/20 mL (5.3 mg/mL), while it is currently under evaluation in Europe and United States for pediatric usage. The dosage schedule based on the patient’s body weight using 20 mg or 250 mg vials of dantrolene sodium is reported in Tables S2 and S3 (Supplementary Files).

The half-life of dantrolene is 4–8 h, and it is highly lipophilic, protein-bound, and undergoes hepatic metabolism [83]. The classical dantrolene formulation has a pH of 9.5 and is associated with injection site reactions, commonly due to extravasation and thrombophlebitis [83]. Side effects of dantrolene include dose-dependent muscle weakness, including respiratory muscles, and hepatotoxicity; mannitol 5% (3000 mg/60 mL) is also present in each vial and may contribute to fluid overload and the development of pulmonary edema [83]. Drug interactions include cardiovascular collapse in combination with calcium channel blockers, especially verapamil, and serotonin antagonist (5HT3-antagonist) antiemetics should be used cautiously [6].

There are no guidelines regarding the availability of sufficient dantrolene for the management of MH crises. The EMHG and the MHAUS recommend that dantrolene be available wherever volatile anesthetics or succinylcholine are used regularly, and 36 vials of dantrolene (or a minimum stock of 3–250 mg vials of RYANODEX^®^) are immediately available. The 36 vials will be enough to treat an MH crisis for 20–30 min in all adult patients of approximately 70 kg. Further dantrolene (to a total of 60 vials within 1 h) will need to be obtained from other sources, and each institution should carefully consider what other sources are available locally and the time taken to obtain them. If additional supplies cannot be obtained within 30 min, they recommend increasing the initial stock supply to 48 vials. They further recommend that remote institutions where more dantrolene cannot be obtained within 1 h should store 60 vials. In large hospitals with more than one operating theatre complex, the stocked dose of dantrolene may be split between the complexes, ensuring that it is readily at hand whenever needed [84].

While the etiologic treatment for malignant hyperthermia is ongoing, maintaining vital functions and correcting biochemical abnormalities is crucial for a good patient outcome. Specifically, the following points should be addressed [8,34,61,78,85,86,87]:

- Temperature management: despite the syndrome’s name, severe hyperthermia is a sign of a delayed diagnosis or management; accordingly, dantrolene is the most appropriate intervention for body temperature control. However, hyperthermia can lead to coagulopathy, irreversible actin-myosin binding, and worsening of acidosis and electrolyte disturbances; therefore, if the body temperature is >38.5–39 °C, active cooling is indicated in addition to turning off heating devices. The pediatric population can very well benefit from surface cooling due to large body surface area in relation to weight; methods include forced air cooling, ice packs near the great arteries (neck, axillae, groins), wet, cold sheets, cooling blankets or pads set at low temperatures (for example used in targeted temperature management). Caution should be made since direct contact with cold objects with the thin skin of a child can cause frostbite. Ice immersion is the most effective method of external cooling, but not applicable in the operating room setting. Cold intravenous fluids administration (4 °C) is a simple and effective second-line cooling method: while fluid replacement can be beneficial for perspiration and to reduce the risk of acute kidney injury, the risk of fluid overload limits this method of cooling; adult guidelines recommend not to exceed 10–20 mL·kg−1, and it is reasonably adequate in the pediatric population. Invasive cooling methods are usually unnecessary in a recognized MH crisis: bladder and gastric lavage are poorly effective, while peritoneal lavage and extracorporeal circulation, albeit effective, require time, equipment, and expertise. Pharmacologic interventions such as acetaminophen or ibuprofen are not effective in this setting. Active cooling should be halted when the core temperature is <38–38.5 °C due to the risk of vasoconstriction and hypothermia in resolving crises.
- Respiratory and metabolic acidosis: minute ventilation should be increased 2–3 times to exhale the excess CO_2_ production by muscle contraction; a normal EtCO_2_ (e.g., 40 mmHg) should be targeted. Metabolic acidosis with base excess < −8 mEq· L^−1^ and pH < 7.2 is treated with sodium bicarbonate 1–2 mEq·kg^−1^.
- Electrolyte disturbances: first-line treatment for hyperkalemia (K^+^ > 5.9 mmol/L or QRS widening) is membrane stabilization with calcium (0.1 mmol·kg^−1^ chloride calcium or 60 mg·kg^−1^ calcium gluconate) and insulin-glucose system for rapid potassium cell entry (e.g., dextrose: 50%, 50 mL with 50 IU insulin for adults or 0.1 insulin·kg^−1^ and dextrose 25%, 2 mL·kg^−1^). Blood glucose should be checked hourly. Other potassium-lowering interventions include sodium bicarbonate, albuterol, furosemide, kayexalate, and hemodialysis.
- Cardiovascular system: arrhythmias should be treated with amiodarone 3 mg·kg^−1^ up to 300 mg. Persistent tachycardia in the absence of hemodynamic compromise can be treated with beta-blockers, while calcium channel blockers should be avoided due to their relevant interaction.
- Renal system: due to rhabdomyolysis and increased creatine kinase, there is a risk of acute kidney injury; guidelines recommend maintaining a high urine output, at least 2 mL·kg^−1^, which can be achieved with cold intravenous fluids, furosemide 0.5–1 mg·kg^−1^, mannitol 1 gr·kg^−1^ (already contained in dantrolene sodium formulation). Urine alkalinization with sodium bicarbonate 1 mEq·kg^−1^·h is also an option.

Criteria for diagnosing the resolution of an MH crisis include decreasing EtCO_2_ or PaCO_2_ for the same minute ventilation or normal EtCO_2_ or PaCO_2_ with normal minute ventilation, decreasing muscle rigidity, decreasing body temperature, and lowering heart rate without arrhythmias. Dantrolene should then be stopped; continuous infusion is not recommended due to the prolongation of weaning from mechanical ventilation. The patient should be transferred to a PICU for at least 24 h since there is a 30% risk of recurrent malignant hyperthermia, requiring further dantrolene administration [88]. Other complications include rhabdomyolysis, acute kidney injury, coagulopathy, and compartment syndrome. Laboratory analysis should include hemoglobin, coagulation panel, renal and hepatic function, creatine kinase, myoglobin, glucose, electrolytes, and arterial blood gases. The prognosis of malignant hyperthermia depends on rapid recognition and early treatment of the crisis, and patients with high muscular mass are at higher risk for adverse outcomes [86,87].

Due to the possibility of a recrudescence of the syndrome within the first 24 h after the initial resolution, patients should be intensively monitored for 48–72 h, and further treatment with dantrolene should be administered.

Conversely, in case of an effective reversal of an MH episode, a continuous infusion or intermittent dantrolene boluses are not recommended, being associated with a high incidence of thrombophlebitis and dose-dependent muscle weakness that may delay weaning from mechanical ventilation [89,90].

The healthcare professional should report the acute MH event to the EMHG or the MHAUS, and the patient and family should be referred for specialized counseling. In addition, medical identification bracelets/necklaces are advisable for these patients, and the importance of notifying the event to health care providers in case of future elective procedures should be emphasized.

## 9. Future Perspectives

Next-generation sequencing facilitates a fast, accurate, and cost-effective genetic analysis, and it is gradually becoming the first-line diagnostic test for genetically heterogenous disorders (such as congenital myopathies) [91]. It caused a “paradigm shift” in MHS diagnostics [72]. As discussed above, RYR1 variants may cause a wide spectrum of muscle diseases, and next-generation sequencing is frequently used in the neuromuscular clinic for RYR1 analysis in patients with an unresolved neuromuscular phenotype [72,92]. This has resulted in a considerable rise in the number of referred patients with a potential risk of MH, even though they have no personal or family history of adverse anesthetic events suspected to be MH.

Bioinformatic model prediction tools need to be improved to classify RYR1 missense variants of unknown significance [91,92]. Future strategies for MH susceptibility diagnostics should focus on the classification of RYR1, CACNA1S, and STAC3 variants utilizing common databases and functional studies in order to prevent unnecessary invasive diagnostic procedures [72].

There are complex variations in genetic and environmental factors underlying the diseases associated with RyR1 dysfunction. Recently, complex models using machine learning techniques and integrating heterogeneous data from different types of tests to diagnose diseases and predict treatment outcomes in a real-world context are being developed [93,94].

## 10. Conclusions

MH still represents a rare but life-threatening disease to which the pediatric population undergoing GA is particularly vulnerable. While genetic predisposition has been linked to MHS, the potential for the involvement of further unknown genes cannot be discounted. Moreover, a number of environmental stressors have also been implicated as risk factors in MHS individuals, but there is as yet no clear consensus from the literature.

An effective prevention policy focused on clearly recognizing the high-risk population, defining perioperative trigger-free hospitalization, and rapid activation of supportive therapy is paramount to avoid adverse outcomes. The incidence of death due to MH has decreased in the last thirty years, but vigilance must be maintained where triggering drugs are administered.

## Figures and Tables

**Table 1:** Neuromuscular weakness classification for HM risk.

Disease	Evidence in Adults	Evidence in Children	Suggested Perioperative Pathway
**Upper motor neurons disease**			
Amyotrophic lateral sclerosis	None	None	standard
**Myelin sheath disease**			
Multiple sclerosis	None	None	Standard
Guillain–Barré syndrome	None	None	Standard
Chronic inflammatory demyelinating polyneuropathy	None	None	Standard
Alexander disease	None	None	Standard
Krabbe disease	None	None	Standard
Adrenoleukodystrophy	None	None	Standard
Neuromyelitis optica spectrum disorders	None	None	Standard
**Neuromuscular junction disease**			
Miastenia gravis	None	None	Standard
**Muscular Dystrophy**			
Duchenne muscular dystrophy	Mild	Mild	Trigger-Free
Congenital muscular dystrophy	Mild	Mild	Trigger-Free
Facioscapulohumeral muscular dystrophy	Mild	Mild	Trigger-Free
Emery–Dreifuss muscular dystrophy	Mild	Mild	Trigger-Free
Becker muscular dystrophy	Mild	Mild	Trigger-Free
**Channel disease**			
Myotonia congenita	None	None	Standard
Hypokalemic periodic paralysis	Strong	Strong	Trigger-Free
Central core disease	Strong	Strong	Trigger-Free
**Cellular Metabolism disease**			
Mitochondrial disease	None	None	Standard
Kearns–Sayre syndrome	None	None	Standard
Glycogen storage disease	None	None	Standard
Lipid storage disorder	None	None	Standard
**Other**			
Neonatal Palsy	None	None	Standard
Traumatic Damage	None	None	Standard

## Supplementary Materials

The following supporting information can be downloaded at: https://www.mdpi.com/article/10.3390/jcm12123869/s1. Table S1: Dosage schedule based on patient’s weight calculated using 20-mg vials of dantrolene reconstituted with 60 mL of sterile water for injection. To calculate mg dosage, multiply patient weight in kg by desired mg·kg^−1^ dose. Multiply the mg dose needed by 60 and divide by 20 for the mL needed. Table S2. Dosage schedule based on patient’s weight calculated using 250-mg vials of dantrolene (Ryanodex^®^) reconstituted with 5 mL of sterile water for injection. To calculate mg dosage, multiply patient weight in kg by desired mg/kg dose. Divide the mg dose by 50 for the mL needed.

## Abbreviations

ACFs	Activated charcoal filters
Ca^2+^	Calcium
CCD	Central core disease
CHCT	Caffeine/halothane contracture test
DHPR	Dihydropyridine receptor
EMHG	European Malignant Hyperthermia Group
ETCO_2_	End-tidal carbon dioxide
GA	General anesthesia
HypoPP	Hypokalemic periodic paralysis
IVCT	In vitro contracture test
MELAS	Mitochondrial encephalomyopathy, lactic acidosis, and stroke-like episodes
MH	Malignant hyperthermia
MHANZ	Malignant Hyperthermia Group of Australia and New Zealand
MHAUS	Malignant Hyperthermia Association of the United States
MHS	Malignant hyperthermia susceptibility
MmD	Multi-mini core disease
MMR	Masseter muscle rigidity
PICU	Pediatric intensive care unit
RyR1	Type 1 ryanodine receptor
SERCA	Sarcoendoplasmic reticulum calcium ATPase
SR	Sarcoplasmic reticulum
TIVA	Total intravenous anesthesia
