# Supplementary material for: Real Evidence and Misconceptions about Malignant Hyperthermia in Children: A Narrative Review
Source: J Clin Med. 2023 Jun 6;12(12):3869. doi: 10.3390/jcm12123869 (PMC10299046; doi:10.3390/jcm12123869)
Supplement: Supplementary file 1 [file jcm-12-03869-s001.zip › jcm-2399670-supplementary.pdf]

Table S1. Dosage schedule based on patient's weight calculated using 20-mg vials of dantrolene reconstituted with 60 mL of sterile water for injection. To calculate mg dosage, multiply patient weight in kg by desired mg.kg^{-1} dose. Multiply the mg dose needed by 60 and divide by 20 for the mL needed.

	dantrolene needed (mg.kg^{-1})	dosage of dantrolene needed (mg)	mL of reconstituted dantrolene to administer	Number of 20 mg vials to reconstitute in 60 ml of sterile water
3	1	3 mg	9 mL	1
5	1	5 mg	15 mL	1
10	1	10 mg	30 mL	1
15	1	15 mg	45 mL	1
20	1	20 mg	60 mL	1
25	1	25 mg	75 mL	2
30	1	30 mg	90 mL	2
50	1	50 mg	150 mL	3
3	2	6 mg	18 mL	1
5	2	10 mg	30 mL	1
10	2	20 mg	60 mL	1
15	2	30 mg	90 mL	2
20	2	40 mg	120 mL	2
25	2	50 mg	150 mL	3
30	2	60 mg	180 mL	3
50	2	100 mg	300 mL	5
3	2.5	7.5 mg	22.5 mL	1
5	2.5	12.5 mg	37.5 mL	1
10	2.5	25 mg	75 mL	2
15	2.5	37.5 mg	112.5 mL	2
20	2.5	50 mg	150 mL	3
25	2.5	62.5 mg	187.5 mL	4
30	2.5	75 mg	225 mL	4
50	2.5	125 mg	375 mL	7
3	5	15 mg	45 mL	1
5	5	25 mg	75 mL	2
10	5	50 mg	150 mL	3
15	5	75 mg	225 mL	4
20	5	100 mg	300 mL	5
25	5	125 mg	375 mL	6
30	5	150 mg	450 mL	8
50	5	250 mg	750 mL	13
3	10	30 mg	90 mL	2
5	10	50 mg	150 mL	3
10	10	100 mg	300 mL	5
15	10	150 mg	450 mL	8
20	10	200 mg	600 mL	10
25	10	250 mg	750 mL	13
30	10	300 mg	900 mL	15
50	10	500 mg	1500 mL	25
3	15	45 mg	135 mL	3
5	15	75 mg	225 mL	4
10	15	150 mg	450 mL	8
15	15	225 mg	675 mL	12
20	15	300 mg	900 mL	15
25	15	375 mg	1125 mL	19

30	15	450 mg	1350 mL	23
50	15	750 mg	2250 mL	38
3	20	60 mg	180 mL	3
5	20	100 mg	300 mL	5
10	20	200 mg	600 mL	10
15	20	300 mg	900 mL	15
20	20	400 mg	1200 mL	20
25	20	500 mg	1500 mL	25
30	20	600 mg	1800 mL	30
50	20	1000 mg	3000 mL	50

Table S2. Dosage schedule based on patient's weight calculated using 250-mg vials of dantrolene (Ryanodex®) reconstituted with 5 mL of sterile water for injection. To calculate mg dosage, multiply patient weight in kg by desired mg/kg dose. Divide the mg dose by 50 for the mL needed.

Patient's weight (kg)	dantrolene needed (mg.kg^{-1})	dosage of dantrolene needed (mg)	mL of reconstituted dantrolene to administer	Number of 250 mg vials to reconstitute in 5 ml of sterile water
3	1	3 mg	0.06 mL	1
5	1	5 mg	0.1 mL	1
10	1	10 mg	0.2 mL	1
15	1	15 mg	0.3 mL	1
20	1	20 mg	0.4 mL	1
25	1	25 mg	0.5 mL	1
30	1	30 mg	0.6 mL	1
50	1	50 mg	1 mL	1
3	2	6 mg	0.12 mL	1
5	2	10 mg	0.2 mL	1
10	2	20 mg	0.4 mL	1
15	2	30 mg	0.6 mL	1
20	2	40 mg	0.8 mL	1
25	2	50 mg	1 mL	1
30	2	60 mg	1.2 mL	1
50	2	100 mg	2 mL	1
3	2.5	7.5 mg	0.15 mL	1
5	2.5	12.5 mg	0.25 mL	1
10	2.5	25 mg	0.5 mL	1
15	2.5	37.5 mg	0.75 mL	1
20	2.5	50 mg	1 mL	1
25	2.5	62.5 mg	1.25 mL	1
30	2.5	75 mg	1.5 mL	1
50	2.5	125 mg	2.5 mL	1
3	5	15 mg	0.3 mL	1
5	5	25 mg	0.5 mL	1
10	5	50 mg	1 mL	1
15	5	75 mg	1.5 mL	1
20	5	100 mg	2 mL	1
25	5	125 mg	2.5 mL	1
30	5	150 mg	3 mL	1
50	5	250 mg	5 mL	1
3	10	30 mg	0.6 mL	1
5	10	50 mg	1 mL	1
10	10	100 mg	2 mL	1
15	10	150 mg	3 mL	1
20	10	200 mg	4 mL	1
25	10	250 mg	5 mL	1
30	10	300 mg	6 mL	2
50	10	500 mg	10 mL	2
3	15	45 mg	0.9 mL	1
5	15	75 mg	1.5 mL	1
10	15	150 mg	3 mL	1
15	15	225 mg	4.5 mL	1
20	15	300 mg	6 mL	2
25	15	375 mg	7.5 mL	2
30	15	450 mg	9 mL	2
50	15	750 mg	15 mL	3

3	20	60 mg	1.2 mL	1
5	20	100 mg	2 mL	1
10	20	200 mg	4 mL	1
15	20	300 mg	6 mL	2
20	20	400 mg	8 mL	2
25	20	500 mg	10 mL	2
30	20	600 mg	12 mL	3
50	20	1000 mg	20 mL	4
